# Evaluation of fitness levels of children with a diagnosis of acute leukemia and lymphoma after completion of chemotherapy and autologous hematopoietic stem cell transplantation

**DOI:** 10.1002/cam4.193

**Published:** 2014-02-12

**Authors:** Antonino Bianco, Antonino Patti, Ewan Thomas, Romilda Palma, Maria Cristina Maggio, Antonio Paoli, Antonio Palma

**Affiliations:** 1 Sport and Exercise Sciences Research Unit, University of PalermoPalermo, Italy; 2 Department of Sciences for Health Promotion and Mother to Child Care “G. D'Alessandro”, University of PalermoPalermo, Italy; 3 Department of Biomedical Sciences, University of PaduaPadua, Italy

**Keywords:** Cancer, fitness, fitness tests, leukemia, lymphoma

## Abstract

The aim of this study was to assess the fitness levels and possible deficits in physical performance in children with a diagnosis of childhood acute leukemia and lymphoma after 10 months of therapy ending through a specific test battery. A total of 58 subjects were enrolled in this study. The experimental group (EG) (7.55 ± 2.43 years; 41.8 ± 16.37 kg; 144.6 ± 10.21 cm) consisted of 18 children with diagnosed leukemia and lymphoma after completion of 10 months of therapy intervention and 40 healthy children who were enrolled in a control group (CG) (7.92 ± 1.78 years; 37.4 ± 12.37 kg; 140.6 ± 12.61 cm). A testing battery including the standing broad jump; the sit-up test; the 4 × 10 m shuttle run test, and the hand grip strength test were administered to both groups. An unpaired *t*-test was adopted to determine differences and the Pearson product moment was administered when appropriate. Results of the EG were generally lower when compared to the CG. Significant differences were identified for the standing broad jump (*P *<* *0.05); 4 × 10 m shuttle run (*P *<* *0.05); hand grip test DX (*P *<* *0.05), and hand grip test SX (*P *<* *0.05). No significant differences were found between the sit-up tests. Pearson product moment correlation revealed a good interaction for all EG participants. Findings suggest that the proposed testing battery could be an appropriate tool to evaluate residual fitness levels in children with previous hematological malignancies. However, our results have to be confirmed with a larger number of participants with the same diagnosis of our EG.

## Introduction

The treatment strategy of the most common childhood acute leukemia and lymphoma consists on a high-dose of chemotherapy and autologous hematopoietic stem cell transplantation (HSCT) 1,2. Scientific innovations have greatly improved cure rates, with 5-year survivals now approaching 80% of treated with HSCT 3,4. However, major concerns have been raised about the treatment and related numerous negative physical and psychological side effects associated with the deterioration of quality of life 5,6. The most frequent complications are: impaired growth and development, cognitive dysfunction, diminished neurological function, cardiopulmonary deficiencies, musculoskeletal complications, and secondary malignancies 7,8. However, children with a diagnosis of childhood hematological malignancies also show decreased levels of fitness and much more sedentary lifestyle compared with their healthy peers 9,10. Recent findings indicate that many survivors have impaired neuromuscular functions which persists post treatment completion 11,12. In particular, children with lymphoblastic leukemia show an increased risk of cardiovascular diseases and obesity as major consequence of their pathology and medical interventions 13–16. This impairment reduces quality of life (QoL) and increases the prevalence of morbidities, with a subsequent decrease in maximal aerobic capacity and reduced maximal strength 15. Recent studies have hypothesized that a physical fitness program following cancer treatment could positively affect additional side effects of this illnesses, such as osteoporosis or decreased muscle tone but also counteract the weight gain often associated with postcancer treatment 17–19. Chang et al. reported decreased levels of fatigue, anxiety, and depression experienced by cancer patients after a 3 week walk training intervention, when compared to a healthy control group (CG) 20. The evaluation of physical fitness has been widely investigated in scientific literature 21–23. Due to their low costs, ease of use, high level of accuracy, and their multiple simultaneous administration capacity, in the last decades, authors have emphasized the importance of field based fitness tests such as the hand grip test, the standing broad jump, the 4 × 10 m shuttle run test and the sit-up test 24.

Therefore, the objective of the current study was the assessment of physical fitness levels in children with previous acute leukemia and lymphoma through a specific fitness test battery 25. Furthermore, we wanted to evaluate the impact of the oncological diseases on children's motor skills and ontogenesis.

## Material and Methods

### Study design and context

We performed an observational case–control study. The 58 subjects that were recruited for the study were divided into two groups. The experimental group (EG) consisted of 18 children with diagnosis of childhood acute leukemia and lymphoma, while the CG consisted of 40 healthy children with similar anthropometric and age characteristics. The EG was recruited from the Pediatric Hematology and Oncology Unit, ARNAS Civico, Di Cristina and Benfratelli Hospitals, Palermo, Italy. Children were enrolled in the study according to the inclusion criteria approved by the ethics committee of the University of Palermo. The study was performed in compliance with the Declaration of Helsinki and the principles of the Italian data protection act (196/2003) were observed. Prior to enrollment, all parents provided informed consent.

### Subjects

Our study contained both genders (Table 1). We recruited 18 children as EG (age: 7.55 ± 2.43 years; weight: 41.8 ± 16.37 kg; height: 144.6 ± 10.21 cm) and 40 children as CG (age: 7.92 ± 1.78 years; weight: 37.4 ± 12.37 kg; height: 140.6 ± 12.61 cm). Children in EG were chosen on the basis of the following criteria: (1) diagnosis of childhood acute leukemia and lymphoma; (2) being in remission; (3) enrolled from 10 to 24 months after remission.

**Table 1:** The table one is providing information regarding the anthropometric characteristics of control group (CG) and the experimental group (EG).

	Control group	Experimental group	*P*
Subjects (*n*)	40	18	
Age (years)	7.92 ± 1.78	7.55 ± 2.43	>0.05
Weight (kg)	37.4 ± 12.37	41.8 ± 16.37	>0.05
Height (cm)	140.6 ± 12.61	144.6 ± 10.21	>0.05

According to Gohar et al.^26^ and following all medical staff directives, a 10-month recovery period is required before the start of a physical activity program or any other social life activity. In order to select the CG we adopted the following inclusion criteria: (1) similar age, weight, height of EG; (2) a similar geographic provenience; (3) not having participated in any regular exercise program. Participants were recruited from a primary school after approval by the dean and a specific anamnesis questionnaire provided by the teachers to the parents.

### Data collection

The anthropometric characteristics of the participants were collected from the same research unit during the period between April 2011 and June 2012 at the Pediatric Hematology and Oncology Unit, ARNAS Civico. Height and weight were measured through a stadiometer (Seca 22 ± 1 mm approximation, Hamburg, Germany). Anthropometric measurements of EG and CG were taken in the hospital; the research unit collected all data during the same period. All tests were administered in a gym during the period between January 2012 and June 2012.

### Method of testing

The specific fitness test battery was administered to CG and EG. The testing battery included the standing broad jump, the hand grip test, the 4 × 10 m shuttle run test and the sit-up test 23,27. All tests were administered following standardized procedures (SOP: standard operating procedures) adopted in previous studies 21–23.

### Statistical analysis

An unpaired *t*-test was adopted to detect significant differences between CG performances and EG performances. A *P* value lower than 0.05 was considered as statistically significant. To evaluate the correlation between the tests, we used the Pearson's correlation coefficient. To perform the analysis, the StatSoft's STATISTICA software (Windows, Vers. 8.0; Tulsa, OK) was used.

## Results

Performance results of the EG were significantly lower compared to the CG: standing broad jump results (CG 121.5 ± 28.44 cm vs. EG 99.78 ± 22.38 cm) showed statistical significant difference (*ρ* < 0.05); 4 × 10 m shuttle run results (CG 14.28 ± 1.50 sec vs. EG 16.04 ± 2.20 sec) showed statistical significant difference (*ρ* < 0.05); sit-up test results (CG 24.58 ± 13.01 reps vs. EG 21.33 ± 16.48 reps) showed no statistical significant difference; hand grip test DX (CG 12.55 ± 4.26 kg vs. EG 9.23 ± 2.96 kg) showed statistical significant difference (*ρ* < 0.05); hand grip test SX (CG 12.21 ± 4.49 kg vs. EG 9.43 ± 3.06 kg) showed statistical significant difference (*ρ* < 0.05) (Tables 1 and 2).

**Table 2:** The table provides a measure of physical fitness levels of both control and experimental group.

	Control group (*N* *=* 40)	Experimental group (*N* *=* 18)	*P*
Standing broad jump (cm)	121.5 ± 28.44	99.78 ± 22.38	<0.05
4 × 10 m shuttle run (sec)	14.28 ± 1.50	16.04 ± 2.20	<0.05
Sit-up test (reps)	24.58 ± 13.01	21.33 ± 16.48	>0.05
Hand grip test DX (kg)	12.55 ± 4.26	9.23 ± 2.96	<0.05
Hand grip test SX (kg)	12.21 ± 4.49	9.43 ± 3.06	<0.05

As we can see children with previous hematological malignancies are showing lower performance indexes.

Pearson analysis of the EG data showed significant correlations between variables: hand grip test of the right hand (DX) (9.2 ± 3.0 kg) versus age (7.5 ± 2.4 years) (*r* = 0.57); hand grip test of the left hand (SX) (9.4 ± 3.1 kg) versus age (7.5 ± 2.4 years) (*r* = 0.56); standing broad jump (99.8 ± 22.4 cm) versus 4 × 10 m shuttle run (16.04 ± 2.2 sec) (*r* = −0.59); standing broad jump (99.8 ± 22.4 cm) versus hand grip test DX (9.2 ± 3.0 kg) (*r* = 0.66); standing broad jump (99.8 ± 22.4 cm) versus hand grip test SX (9.4 ± 3.1 kg) (*r* = 0.68); 4 × 10 m shuttle run (16.04 ± 2.2 sec) versus sit-up test (21.3 ± 16.5 reps) (*r* = −0.71); standing broad jump (99.8 ± 22.4 cm) versus sit-up test (21.3 ± 16.5 reps) (*r* = 0.49); and hand grip test DX (9.2 ± 3.0 kg) versus hand grip test SX (9.4 ± 3.1 kg) (*r* = 0.95). Pearson's results coming from CG data as expected showed a similar trend. Hand grip test DX (12.5 ± 4.3 kg) versus age (7.9 ± 1.8 years) (*r* = 0.71); hand grip test SX (12.2 ± 4.5 kg) versus age (7.9 ± 1.8 years) (*r* = 0.74), standing broad jump (121.5 ± 28.4 cm) versus 4 × 10 m shuttle run (14.3 ± 1.5 sec) (*r* = −0.65); standing broad jump (121.5 ± 28.4 cm) versus hand grip test DX (12.5 ± 4.3 kg) (*r* = 0.58); standing broad jump (121.5 ± 28.4 cm) versus hand grip test SX (12.2 ± 4.5 kg) (*r* = 0.65); 4 × 10 m shuttle run (14.3 ± 1.5 sec) versus sit-up test (24.6 ± 13.0 reps) (*r* = −0.65); standing broad jump (121.5 ± 28.4 cm) versus sit-up (24.6 ± 13.0 reps) (*r* = 0.44); and hand grip test DX (12.5 ± 4.3 kg) versus hand grip test SX (12.2 ± 4.5 kg) (*r* = 0.91).

## Discussion

Our results are confirmed by previously published studies 28–32 as the present research demonstrates that children with previous hematological malignancies have lower physical fitness levels when compared to their healthy peers (Table 2). In agreement with Taskinen et al. 33, our results further demonstrate that there are no contraindications to start a physical activity program. The novelty of our approach consisted in the administration of a field-based assessment methodology. Although the pathology in the EG is in remission, secondary side effects from the chemotherapy, affecting multiple organ systems may be present. Chemotherapeutic agents have well-documented toxic effects on different organs and can adversely affect lungs, heart, and muscles 34–36. Corticosteroid therapy, in particular protocols with dexamethasone, is associated with obesity or overweight conditions as an early or late side effect 37. Oeffinger et al. 7. reported that one-third of childhood cancer survivors have severe or life-threatening medical complications 30 years post diagnosis 7,38,39. The literature suggests an adverse influence of physical performance capacities during adulthood in the presence of neuromuscular deficits 12,40. Scientists and medical practitioners, during the last decade have shown a growing interest not only in the illness survival but also in the QoL of survivors. Consequently, new tools and new strategies for the evaluation of physical fitness in children with previous malignancies are needed 22,25. The present study used a specific test battery comprising of validated field-based fitness tests to gain a better understanding of physically related consequences of previous malignancies. Fitness tests are usually applied in sporting context. This is the first study which has adopted these field-based tests to further help “survivors” improving their QoL. In our experience, the testing battery showed to be simple and fast to administer, to be highly versatile, to be inexpensive, and to be reliable 25. In conclusion, children with previous malignancies show a lower level of physical fitness but appear to be physically ready to engage in regular exercise interventions. Ideally, our results require further support by a large-scale study.

## Conflict of Interest

None declared.
